# A Guide to the Practical Examination of Urine

**Published:** 1903-02

**Authors:** 


A Guide to the Practical Examination of Urine. For the use of Students and Physicians. By James Tyson, M. D., Professor of Medicine in the University of Pennsylvania, and Physician to the Hospital of the University. Tenth edition, revised and corrected. Philadelphia: P. Blakiston's Son & Co. 1902. [Price, $1.50 ]

This is the tenth edition of the best work on uranalysis which has ever been published in manual form. Dr. Tyson has revised and corrected the work and added many points of value to the uranalyst in his daily work. As it stands now, it is quite difficult to see how it can possibly be improved upon by any further revision, yet we may expect another in the future, for Dr. Tyson keeps this work abreast the latest developments in the branch of which he is master. The popularity of the book is attested by the demand which has necessitated this new edition. It is freely and intelligently illustrated. The general practitioner who makes his own examinations cannot err if he follows Tyson. N. W. W.

